# A Novel Function of Glycerol Kinase Alleviates LPS-Induced Inflammatory Responses by the p38/STAT3 Pathway and Mitigates ROS Generation in Kupffer Cells

**DOI:** 10.3390/antiox14101174

**Published:** 2025-09-26

**Authors:** Yanfei Li, Xu Zhang, Danping Wang, Guoqiang Fan, Xiaojing Yang

**Affiliations:** 1 Key Laboratory of Animal Physiology & Biochemistry, Nanjing Agricultural University, Nanjing 210095, China; 2021207010@stu.njau.edu.cn (Y.L.); fan.gq@njau.edu.cn (G.F.); 2 MOE Joint International Research Laboratory of Animal Health and Food Safety, Nanjing Agricultural University, Nanjing 210095, China

**Keywords:** glycerol kinases, Kupffer cells, inflammation, ROS, MAPK signaling pathway

## Abstract

Kupffer cells (KCs), the predominant resident macrophages in the liver, exhibit an inflammatory activation state that is pathologically linked to various hepatic disorders. Studies have shown that macrophages undergo metabolic reprogramming under inflammatory conditions, and the expressions of glucose and lipid metabolism-related factors change significantly. However, glycerol kinase (GK), as a related factor that links glycolipid metabolism, the role of GK in inflammatory conditions, and its mechanism have not been reported. The aim of the present study was to explore the role of GK in the inflammatory response of KCs. LPS challenge induced marked dysregulation of glucose and lipid metabolic profiles, accompanied by a significant elevation in GK expression in pro-inflammatory KCs. GK significantly decreased the expression of pro-inflammatory factors in LPS-treated KCs. Further studies found that GK can alleviate the level of LPS-stimulated reactive oxygen species (ROS) and the expression of antioxidant factors. Meanwhile, the results showed that GK alleviates LPS-induced KCs inflammation through inhibiting the p38/STAT3 signaling pathway. The results of this study are the first to reveal that GK may alleviate Kupffer cells’ inflammatory responses by inhibiting the p38/STAT3 signaling pathway and mitigating LPS-induced ROS generation. The findings provide a potential reference for future development of drugs targeting GK to prevent KCs inflammation and even liver damage.

## 1. Introduction

Immune cells are involved in or associated with immune responses, most of which are relatively static in a stable state, but all have the ability to respond quickly to infection, inflammation, and other disturbances. As an important immune cell, macrophages undergo metabolic remodeling to adapt to their functional changes when switching between resting and active states [1,2]. More and more studies have shown that the metabolic level of macrophages affects their immune function [3,4]. During the inflammation activation phase, macrophages increase glycolysis, significantly upregulate the key enzyme in the glycolytic pathway, hexokinase [5,6], and reduce mitochondrial oxidative phosphorylation [7,8]. Studies have shown that pro-inflammatory M1 macrophages mainly rely on glycolytic metabolism, and M2 macrophages mainly rely on oxidative phosphorylation to provide energy [9,10]. Studies have shown that significant changes occur in lipid metabolism-related factors after the activation of M1-type macrophages, indicating that lipid metabolic reprogramming is involved in the polarization process of macrophages [11,12]. Under the pro-inflammatory phenotype activated by LPS, the level of glycolysis is significantly increased to support cell lactic acid biosynthesis [13] and lipid droplet accumulation [14]. Since more and more studies have shown the important role of metabolism in immune cell inflammation, targeting metabolism-related factors to regulate the metabolic way of immune cells has important significance for the level of immune cell inflammation. As immune cells of the liver, Kupffer cells (KCs) secrete cytokines to accelerate the removal of pathogens and resist the invasion of various pathogens after the liver is stimulated [15]. Targeted regulation of KCs is important for the treatment of inflammation. However, at present, there are few studies on the metabolic reprogramming of KCs in the case of inflammation, and the specific mechanism of targeting the metabolic-related factors of KCs to regulate their metabolic pathways to regulate inflammation needs to be further discovered.

Glycerol kinase (GK) belongs to the FGGY kinase family and is an enzyme that regulates glycerol uptake and metabolism, catalyzes ATP phosphorylation of glycerol to produce glycerol-3-phosphate (G3P) and ADP, and dehydrogenates G3P to produce dihydroxyacetone phosphate (DHAP), which can be used as a raw material for gluconeogenesis to synthesize glucose or release energy along the pathway of glucose metabolism. G3P can also participate in the synthesis of triglycerides (TGs). It plays an important role in glycolipid metabolism. Research shows that GK deficiency can lead to metabolic disorders and various diseases, affecting growth and development. GK KO mice died at 3–4 days after birth and were accompanied by hypertriglyceridemia and elevated plasma free fatty acid contents [16,17,18]. GK deficiency is associated with Duchenne muscular dystrophy and congenital renal hypoplasia as manifestations of Xp21 continuous gene deficiency syndrome [19,20]. Dexamethasone treatment can significantly activate the activity of GK in brown fat, increase the content of glycerol kinase, promote the synthesis of G3P to TG, and promote the whiteness of brown fat [21]. In addition to its role as a glycerol metabolic kinase, GK also has the moonlighting enzyme activity of other moonlighting enzymes to regulate the level of glucose and lipid metabolism [22,23,24,25,26]. GK, similar to hexokinase, is located on the surface of the mitochondrial membrane and binds to mitochondrial membrane proteins to play an important role [27,28]. These results indicate that GK plays an important role in the regulation of cell metabolism levels; however, the regulatory effect of GK on macrophages in inflammatory conditions and its specific mechanism have not been reported.

In this study, we explored the metabolic changes in the inflammatory response of Kupffer cells and the role and mechanism of GK, a key factor in glycolipid metabolism, in the LPS-induced inflammatory response of Kupffer cells.

## 2. Materials and Methods

### 2.1. Cell Culture

KCs were purchased from the BeNa Culture Collection. KCs were cultured in a growth medium composed of 1640 medium (Wisent) and 10% fetal bovine serum supplemented with penicillin and streptomycin at 37 °C in a 5% CO_2_ atmosphere. GK siRNA and negative control FAM (NC), and an overexpression plasmid and a negative control plasmid (NC) (GenePharma, Shanghai, China), were transfected into KCs by the JetPRIME transfection reagent, and then LPS (100 ng/mL) was added to the medium for 6 h. The p38 inhibitor adezmapimod (HY-10256, MCE, Belleville, NJ, USA) was added 2 h before the LPS was added.

### 2.2. Transcriptomic Analysis

KCs were treated with LPS (1 μg/mL) for 24 h. The total RNA of KCs was isolated and purified by TRIzol reagent (Invitrogen, Carlsbad, CA, USA). The RNA purity and content of samples were detected by a NanoDrop ND-1000 (NanoDrop Technologies, Wilmington, DE, USA). The mRNA library was constructed and sequenced by BioNovoGene Co., Ltd. (Suzhou, China) and LC-Bio Technology CO., Ltd. (Hangzhou, China). mRNA expressions were analyzed by the R packages edgeR or DESeq2.

### 2.3. Metabolomic Analysis

KCs were treated with LPS (1 μg/mL) for 24 h. The sample was analyzed by LC-MS/MS mass spectrometry, and the data analysis was processed by Gene Denovo Biotechnology Co., Ltd. (Guangzhou, China). The operation is described as follows: Firstly, the treated KCs were collected into 1.5 mL EP tubes and then frozen with liquid nitrogen and extracted by ultrasonic lysis. The samples were separated by an Agilent 1290 Infinity LC ultra-high performance liquid chromatography (UHPLC) HILIC column. An AB Triple TOF 6600 spectrometer was used to collect the primary and secondary spectral information of the samples. The ProteoWizard (v3.0.6428) software converted the original data into an MzML format, and the XCMS program (online 3.7.1) was used for peak alignment, retention time correction, and peak area extraction.

### 2.4. Isolation of Primary KCs

For the primary KCs’ isolation, firstly, the liver was perfused with 10 mL of phosphate-buffered saline and then digested with 0.1% type IV collagenase. Following digestion, the liver homogenate was filtered through a 75 μm stainless-steel wire mesh to remove undigested tissue. The cell suspension was centrifuged at 50 g (Eppendorf 5810 R, Germany) for 5 min at 4 °C. The top suspension was separated with 60% Percoll and then centrifuged at 2500 g for 25 min. The darker layer in the middle comprised KCs.

### 2.5. Total Protein Extraction and Western Blot Analysis

Total protein was extracted from KCs using RIPI buffer containing the complete EDTA-free and PhosSTOP protease inhibitor cocktail. Protein concentration was determined following the BCA Protein Assay kit protocols. About 30–50 μg of protein was used by 10% SDS pages, and then the gels were transferred to a nitrocellulose membrane. The antibodies used in the Western blot analysis are listed in Table 1. Tannon-5200 was used for capturing the images. The Image J software (version 1.47) was used to analyze the bands’ density. Tubulin-α was used for reference control proteins. The antibody information is shown in Table 1.

### 2.6. Total RNA Isolation and Quantitative Polymerase Chain Reaction (PCR)

Total RNA was isolated from KCs using the TRIzol reagent. An amount of 1 ug of RNA was reverse-transcribed to cDNA following the Transcription Master Kit (Vazyme, Nanjing, China). Diluted cDNA (1:20, *v*/*v*) was used for qPCR with the Mx3000P Real-Time Polymerase Chain Reaction (PCR) System (Stratagene Inc., La Jolla, CA, USA). PPIA was chosen as the reference gene. The qPCR primer sequences are listed in Table 2.

### 2.7. Fluorescence Microscopy

The KCs were stained with ROS (Beyotime, Shanghai, China), PI (Coolaber Science & Technology Co., Ltd., Beijing, China), and Mititracker (Invitrogen, Carlsbad, CA, USA) at 37 °C. Subsequently, these cells were stained with DAPI for 5 min and observed by fluorescent microscopy.

The KCs were fixed with 4% paraformaldehyde for 10 min. The samples were soaked in Tris-buffered saline containing 0.3% Triton X-100 for 1 h, blocked with 10% BSA, and incubated with the ASC antibody (Abcam, Cambridgeshire, UK), GK antibody (Abcam, Cambridgeshire, UK), and NLRP3 antibody (Bioworld, Nanjing, China) overnight at 4 °C and then with the secondary antibody. The cell nuclei were dyed with DAPI.

### 2.8. Flow Cytometry

The KCs were incubated with ROS (Beyotime, Shanghai, China) at 37 °C. Data were acquired by flow cytometry with the BD FACSVerse (BD Biosciences, San Jose, CA, USA) and analyzed with the BD FACSuite.

### 2.9. Detection of MDA and GSH Contents

The GSH and MDA contents in the KCs were detected according to the kit instructions (MDA: Solarbio; GSH: Jiancheng).

### 2.10. Statistical Analysis

All statistical analyses were performed using the Prism 8 software (GraphPad Software Inc., La Jolla, CA, USA), and the results are presented as means ± SEMs. Sample *n* = 3 indicates that we used three independent test samples. Differences were detected using a two-tailed *t*-test. * indicates comparison with the CON group or the NC group, ^#^ indicates comparison with the NC + LPS group. * and ^#^ indicate *p*-value < 0.05, ** and ^##^ indicate *p*-value < 0.01, and *** and ^###^ indicate *p*-value < 0.001.

## 3. Results

### 3.1. LPS Triggers Metabolic Reprogramming of KCs and Is Accompanied by Significant Upregulation of GK

**Figure 1:**
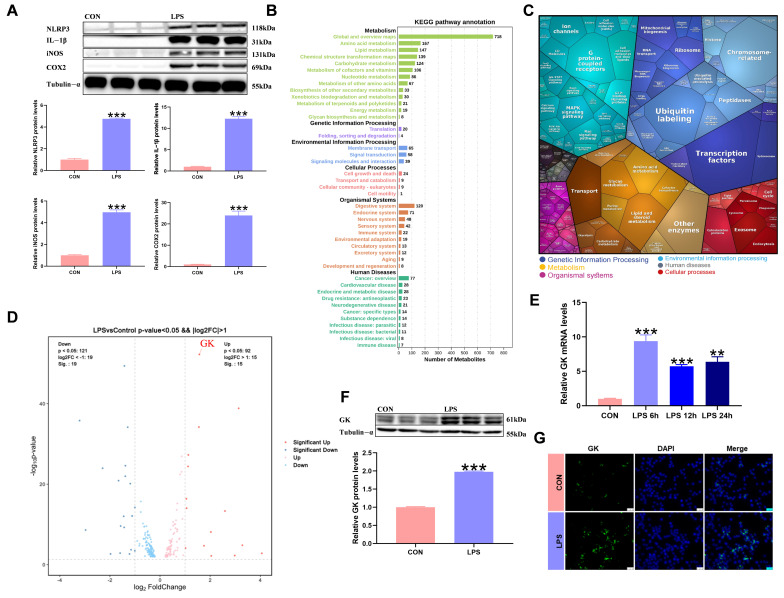
LPS triggers the metabolic reprogramming of KCs and is accompanied by significant upregulation of GK. (**A**) Inflammation-related factors’ protein expression in LPS-stimulated KCs. (**B**) Statistical map of metabolomic KEGG. (**C**) Statistical map of transcriptomic KEGG. (**D**) Volcanic map showing that GK was the significant difference between glucose and lipid metabolism genes in LPS-stimulated KCs. (**E**) GK mRNA expression in LPS-stimulated KCs for 6 h, 12 h, and 24 h. (**F**) GK protein expression in LPS-stimulated KCs for 6 h. (**G**) Immunofluorescence staining for GK expression in LPS-stimulated KCs for 6 h. DAPI was used to visualize nuclei. Scale bars represent 25 μm (*n* = 3). In the graph, the data represent the means ± SEMs; ** indicated *p* < 0.01, *** indicated *p* < 0.001; and *n* = 3.

LPS treatment in KCs significantly increased inflammation-related factors’ expression (Figure 1A). Metabolic reprogramming usually occurs when immune cells are polarized. Metabolomic KEGG pathway analysis showed that metabolism-related pathways were changed after LPS treatment of KCs (Figure 1B). In order to explore the metabolic regulators induced by LPS-treated KCs, transcriptomic KEGG pathway analysis of LPS-treated KCs revealed significant changes related to metabolism (Figure 1C), and studies have shown that LPS treatment of KCs can cause changes in glycolipid metabolism-related factors. Volcanic analysis of factors related to glucose and lipid metabolism showed that GK expression increased significantly (Figure 1D). LPS treatment of KCs for 6 h, 12 h, and 24 h resulted in a significant increase in GK gene expression (Figure 1E). The expression and fluorescence intensity of the GK protein were significantly increased after 6 h of KCs treated with LPS (Figure 1F,G).

### 3.2. GK Improved the LPS-Induced Inflammatory Response of KCs

**Figure 2:**
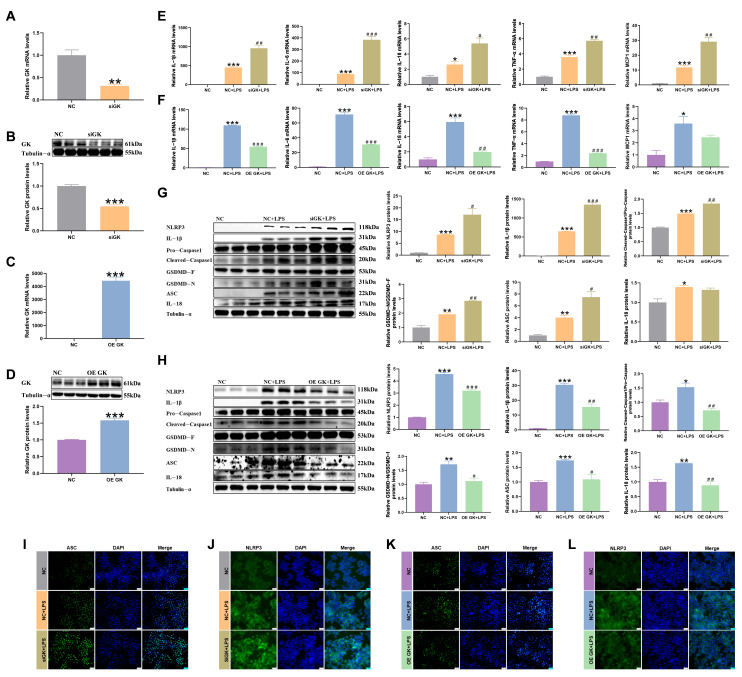
GK improved the LPS-induced inflammatory response of KCs. (**A**) GK gene expression after siRNA treatment in KCs. (**B**) GK protein expression after siRNA treatment in KCs. (**C**) GK gene expression after OE GK treatment in KCs. (**D**) GK protein expression after OE GK treatment in KCs. (**E**) Gene expression of inflammation-related factors after siGK treatment in KCs. (**F**) Gene expression of inflammation-related factors after OE GK treatment in KCs. (**G**) Protein expression of inflammation-related factors after siGK treatment in KCs. (**H**) Protein expression of inflammation-related factors after OE GK treatment in KCs. (**I**) Immunofluorescence of ASC after siGK treatment in KCs (blue: DAPI; green: ASC). Scale bars represent 25 μm. (**J**) Immunofluorescence of NLRP3 after siGK treatment in KCs (blue: DAPI; green: NLRP3). Scale bars represent 25 μm. (**K**) Immunofluorescence of ASC after OE GK treatment in KCs (blue: DAPI; green: ASC). Scale bars represent 25 μm. (**L**) Immunofluorescence of NLRP3 after OE GK treatment in KCs (blue: DAPI; green: NLRP3). Scale bars represent 25 μm. In the graph, the data represent the means ± SEMs; * *p* < 0.05 compared with the NC group; ^#^ *p* < 0.05 compared with the NC + LPS group; ** and ## indicated *p*-value < 0.01, *** and ### indicated *p*-value < 0.001; and *n* = 3.

Next, in order to further study the role of GK in inflammation, siRNA and an overexpression plasmid of GK were used to influence the expression of GK to explore its role in the inflammatory response. siGK significantly decreased the GK mRNA and protein expression, and the overexpression plasmid of GK increased the GK mRNA and protein expression, indicating that siRNA expression and overexpression of GK were successful (Figure 2A–D).

In this result, LPS increased the inflammation-related factors, mRNA, and protein expressions. The results showed that the expressions of the inflammation-related factors IL-1β, IL-6, IL-18, TNF-α, and MCP1 mRNA were increased in the siGK + LPS group compared with the NC + LPS group, and IL-1β, IL-6, IL-18, and TNF-α were decreased in the OE GK + LPS group compared with the NC + LPS group (Figure 2E,F). At the same time, the protein expressions of the inflammation-related factors IL-1β, caspase1, GSDMD, ASC, and NLRP3 were increased in the siGK + LPS group compared with the NC + LPS group, and IL-1β, caspase1, GSDMD, ASC, IL-18, and NLRP3 were decreased in the OE GK + LPS group compared with the NC + LPS group (Figure 2G,H). ASC and NLRP3 fluorescence also increased in the siGK + LPS group compared with the NC + LPS group, and it decreased in the OE GK + LPS group compared with the NC + LPS group (Figure 2I–L).

### 3.3. GK Inhibited iNOS and COX2 Expression in LPS-Stimulated KCs

**Figure 3:**
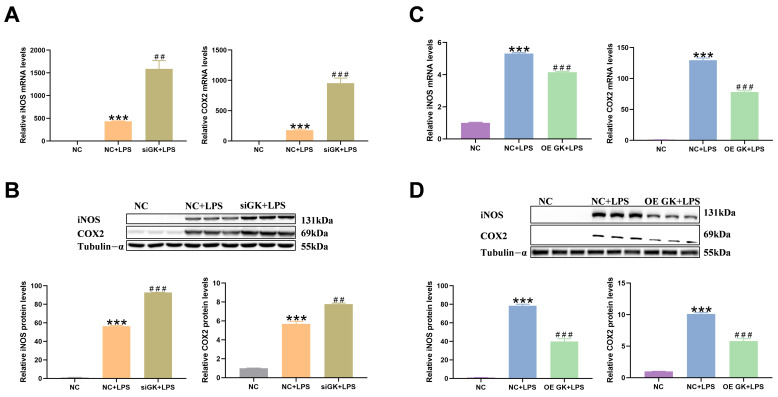
GK inhibited the iNOS and COX2 expression in LPS-stimulated KCs. (**A**) Gene expression of iNOS and COX2 after siGK treatment in KCs. (**B**) Protein expression of iNOS and COX2 after siGK treatment in KCs. (**C**) Gene expression of iNOS and COX2 after OE GK treatment in KCs. (**D**) Protein expression of iNOS and COX2 after OE GK treatment in KCs. In the graph, the data represent the means ± SEMs; ^##^ indicated *p*-value < 0.01, *** and ^###^ indicated *p*-value < 0.001; and *n* = 3.

Two enzymes, iNOS and COX2, were also significantly elevated in the level of inflammation and could be used as markers of inflammation. In this study, we tested the iNOS and COX2 mRNA and protein expression. LPS markedly increased the iNOS and COX2 mRNA and protein expressions. The siGK + LPS group showed significantly increased iNOS and COX2 expressions compared with the NC + LPS group. And OE GK markedly decreased the iNOS and COX2 expression in the OE GK + LPS group compared with the NC + LPS group (Figure 3A–D).

### 3.4. GK Significantly Decreased Apoptosis Level of KCs

**Figure 4:**
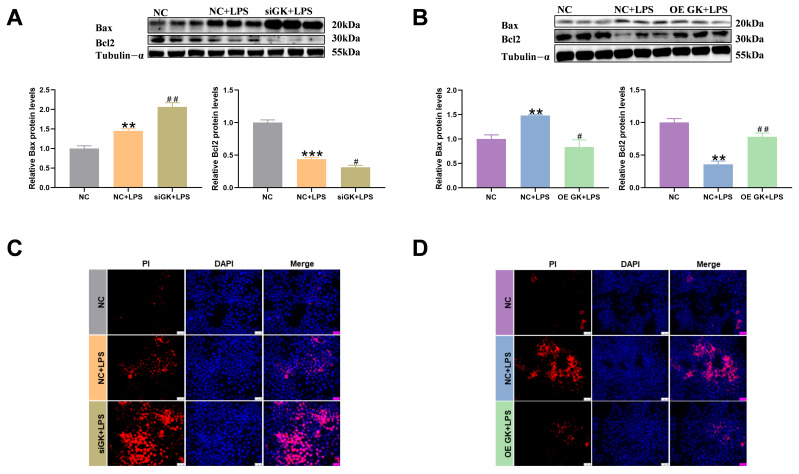
GK significantly decreased the apoptosis level of KCs. (**A**) Protein expression of Bax and Bcl2 after siGK treatment in KCs. (**B**) Protein expression of Bax and Bcl2 after OE GK treatment in KCs. (**C**) PI staining after siGK treatment in KCs (blue: DAPI; red: PI). Scale bars represent 25 μm. (**D**) PI staining after OE GK treatment in KCs (blue: DAPI; red: PI). Scale bars represent 25 μm. In the graph, the data represent the means ± SEMs; ^#^ *p* < 0.05 compared with the NC + LPS group; ** and ^##^ indicated *p*-value < 0.01, *** indicated *p*-value < 0.001; and *n* = 3.

Studies have shown that LPS-induced inflammation is usually accompanied by apoptosis. In this article, we examined apoptosis-related indicators. Bax and Bcl2 are related to apoptosis. Bax can promote apoptosis, while Bcl2 can inhibit apoptosis. In this study, LPS induced Bax expression and decreased Bcl2 expression. The siGK + LPS group increased the Bax level and decreased the Bcl2 expression compared with the NC + LPS group (Figure 4A). The OE GK + LPS group showed a markedly decreased Bax level and increased Bcl2 expression compared with the LPS group (Figure 4B). The PI fluorescence results showed that LPS increased the apoptosis level, and siGK further increased PI fluorescence in the siGK + LPS group compared with the NC + LPS group (Figure 4C). Meanwhile, OE GK further decreased PI fluorescence in the OE GK + LPS group compared with the NC + LPS group (Figure 4D).

### 3.5. GK Significantly Alleviates Oxidative Stress of KCs Induced by LPS

**Figure 5:**
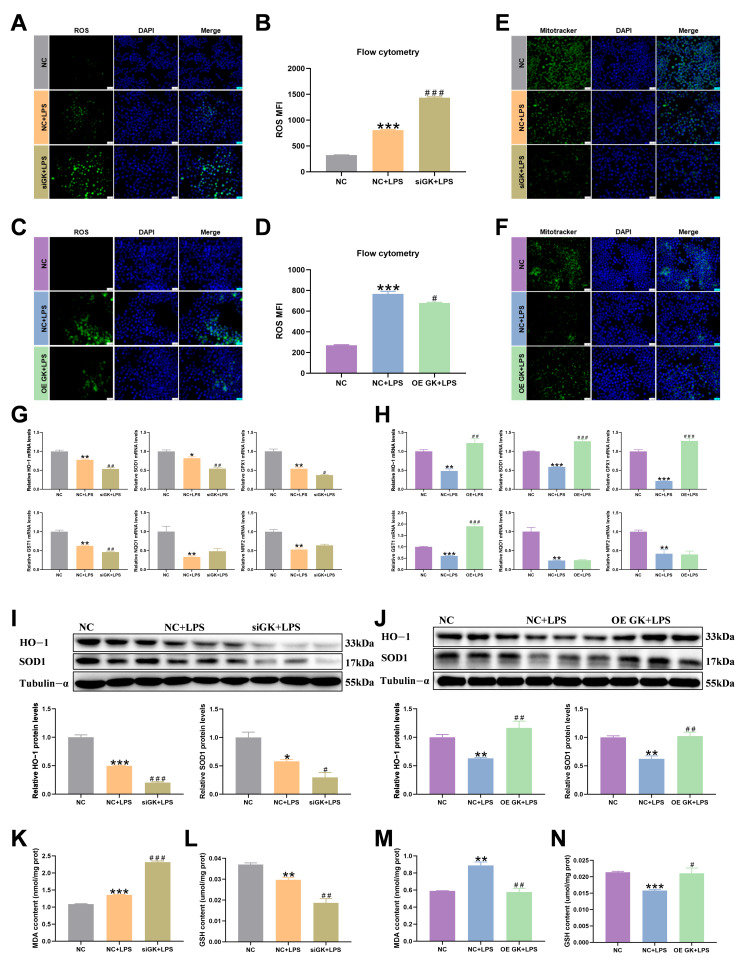
GK significantly alleviates the oxidative stress of KCs induced by LPS. (**A**) Fluorescence intensity of ROS after siGK treatment in KCs (blue: DAPI; green: ROS). Scale bars represent 25 μm. (**B**) ROS level detected by flow cytometry after siGK treatment in KCs. (**C**) Fluorescence intensity of ROS after OE GK treatment in KCs (blue: DAPI; green: ROS). Scale bars represent 25 μm. (**D**) ROS level detected by flow cytometry after OE GK treatment in KCs. (**E**) Mitotracker staining after siGK treatment in KCs (blue: DAPI; green: Mitotracker). Scale bars represent 25 μm. (**F**) Mitotracker staining after OE GK treatment in KCs (blue: DAPI; green: Mitotracker). Scale bars represent 25 μm. (**G**) Gene expression of antioxidants after siGK treatment in KCs. (**I**) Gene expression of antioxidants after OE GK treatment in KCs. (**H**) Protein expression of HO-1 and SOD1 after siGK treatment in KCs. (**J**) Protein expression of HO-1 and SOD1 after OE GK treatment in KCs. (**K**) MDA content after siGK treatment in KCs. (**L**) GSH content after siGK treatment in KCs. (**M**) MDA content after OE GK treatment in KCs. (**N**) GSH content after OE GK treatment in KCs. In the graph, the data represent the means ± SEMs; * *p* < 0.05 compared with the NC group; ^#^ *p* < 0.05 compared with the NC + LPS group; ** and ^##^ indicated *p*-value < 0.01, *** and ^###^ indicated *p*-value < 0.001; and *n* = 3.

Inflammatory conditions are often accompanied by oxidative damage to cells. We detected the relevant indicators of cellular oxidation reaction. The flow cytometry and fluorescence intensity results of ROS showed that the ROS level was increased in the NC + LPS group compared with the NC group, and siGK significantly increased the ROS level in the siGK + LPS group compared with the NC + LPS group (Figure 5A,B), and the OE GK + LPS group exhibited a decreased ROS level compared with the NC + LPS group (Figure 5C,D). Mitotracker fluorescence staining showed that siGK + LPS increased mitochondrial damage compared with the NC + LPS group, and the OE GK + LPS group exhibited decreased mitochondrial damage compared with the NC + LPS group (Figure 5E,F). Next, we tested the antioxidant-related factors’ expression. LPS decreased the antioxidant-related gene expression, and the siGK + LPS group showed further decreased oxidative stress compared with the NC + LPS group, while the OE GK + LPS group showed significantly increased antioxidant-related gene expression compared with the NC + LPS group (Figure 5G,H). LPS significantly decreased the protein expression of the antioxidant-related factors HO-1 and SOD1. The siGK + LPS group showed further decreased SOD1 and HO-1 protein expression compared with the NC + LPS group, and the OE GK + LPS group showed significantly increased SOD1 and HO-1 protein expression compared with the NC + LPS group (Figure 5I,J). Next, we detected the MDA and GSH contents and found that LPS increased the MDA content and decreased the GSH content, siGK further increased the MDA content and decreased the GSH content in the siGK + LPS group compared with the NC + LPS group (Figure 5K,L), and OE GK decreased the MDA content and increased the GSH content in the OE GK + LPS group compared with the NC + LPS group (Figure 5M,N).

### 3.6. GK Influences Inflammation in KCs via Inhibiting PKCε/p38/STAT3 Pathway

**Figure 6:**
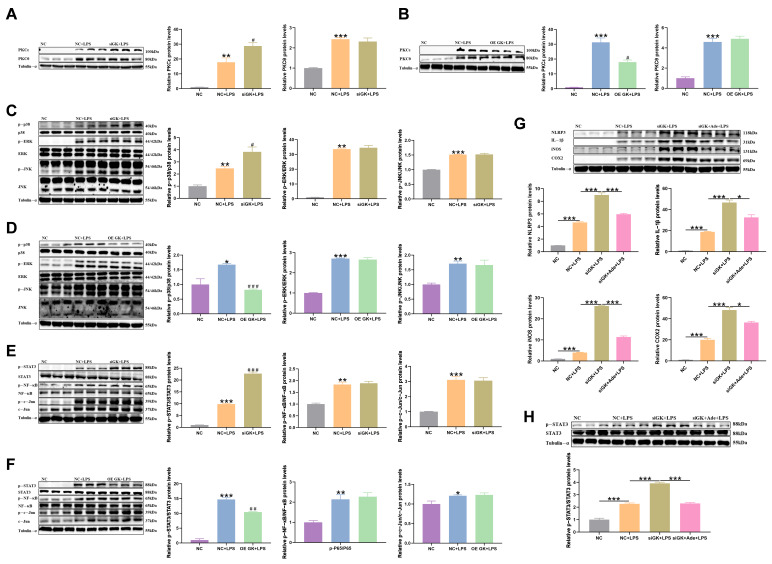
GK influences inflammation in KCs via inhibiting the PKCε/p38/STAT3 pathway. (**A**) PKC protein expression after siGK treatment in KCs. (**B**) PKC protein expression after OE GK treatment in KCs. (**C**) MAPK protein expression after siGK treatment in KCs. (**D**) MAPK protein expression after OE GK treatment in KCs. (**E**) STAT3, NF-κB, and c-Jun protein expressions after siGK treatment in KCs. (**F**) STAT3, NF-κB, and c-Jun protein expressions after OE GK treatment in KCs. (**G**) IL-1β, NLRP3, iNOS, and COX2 protein expressions after p38 inhibitor treatment in KCs. (**H**) STAT3 protein expression after p38 inhibitor treatment in KCs. In the graph, the data represent the means ± SEMs; * *p* < 0.05 compared with the NC group; ^#^ *p* < 0.05 compared with the NC + LPS group; ** and ^##^ indicated *p*-value < 0.01, *** and ^###^ indicated *p*-value < 0.001; and *n* = 3.

PKCε protein expression was significantly increased in the siGK + LPS group compared with the NC + LPS group (Figure 6A), and PKCε protein expression in the OE GK + LPS group was decreased compared with the NC + LPS group (Figure 6B). Next, the expression level of MAPK, a downstream inflammatory regulator of PKC, was detected. LPS increased MAPK protein expression, but siGK only increased the p-p38 expression in the siGK + LPS group compared with the NC + LPS group, and OE GK decreased the p-p38 expression in the OE GK + LPS group compared with the NC + LPS group (Figure 6C,D). The MAPK downstream factors NF-κB, STAT3, and c-Jun also increased in the NC + LPS group compared with the NC group, but GK only decreased the p-STAT3 expression (Figure 6E,F). Next, the p38 inhibitor SB203580 (adezmapimod (Ade)) was used to treat KCs to further confirm the role of p38 in the siGK-aggravated effect on inflammation. The results showed that the p38 inhibitor markedly decreased the protein expressions of the inflammation-related factors IL-1β, NLRP3, iNOS, and COX2. The p38 inhibitor decreased the p-STAT3 protein expression in the siGK + Ade + LPS group compared with the siGK + LPS group (Figure 6G,H).

## 4. Discussion

As an important macrophage in the liver, KCs play an important role in liver immunity, and their inflammatory response has an important impact on the occurrence and development of liver diseases. Targeting KCs is of great significance for the treatment of liver diseases [29,30]. Increasing evidence indicates significant changes in glucolipid metabolic flux in activated macrophages, accompanied by metabolic reprogramming of glucose and lipid metabolism, and reveals specific mechanisms that mitigate LPS-induced inflammatory responses by regulating glucose and lipid metabolism levels [2,31]. In this study, transcriptomic analysis of factors related to glucose and lipid metabolism found that GK expression changed significantly among factors related to glucose and lipid metabolism. And LPS significantly increased the expression of the GK protein and mRNA in KCs, indicating that GK plays an important role in the LPS-induced inflammatory response.

The expression of inflammatory factors plays an important role in the occurrence and development of inflammation. NLRP3 expression was significantly increased in the inflammatory response. NLRP3 can promote the mature secretion of caspase1, and IL-1β promotes inflammatory responses [32,33]. In this study, GK significantly decreased the expression of NLRP3 and IL-1β in LPS-induced KCs. Studies have reported that IL-6, IL-18, and TNF are also important pro-inflammatory factors [34,35]. In this experiment, its gene expression was also significantly changed after siGK and OE GK treatment in KCs. GK expression significantly influenced the expression of LPS-induced IL-1β, IL-6, IL-18, and TNF-α inflammatory factor genes. In addition, two enzymes, COX2 and iNOS, play an important role in mediating the inflammatory process [36,37]. The upregulated expression of iNOS and COX2 is associated with inflammatory diseases and various cachexias [38,39]. The results of this experiment showed that GK significantly decreased the gene and protein expression of iNOS and COX2. Therefore, these results suggest that GK influences the LPS-induced inflammatory response by decreasing the production of inflammatory mediators in KCs.

In addition, cellular inflammation usually leads to cell damage and death. Studies have shown that PI staining, as a detection method for cell death, can distinguish living cells from dead cells, and the inflammatory response can significantly affect cell death and affect the fluorescence intensity of PI [40,41]. As the marker proteins of apoptosis, Bax and Bcl2 play an important role in regulating cell damage. Studies have shown that their expression changes significantly under inflammatory conditions [42,43,44]. After the absence of GK was reported in previous studies, it was found that the expression of apoptosis-related factors was significantly affected by omics analysis, indicating that GK was related to apoptosis [45]. In this study, the PI fluorescence intensity showed that GK significantly decreased apoptosis induced by LPS, and GK decreased the expression of apoptosis-related factors, such as Bax, and increased Bcl2 expression.

ROS play an important role in cell growth and differentiation, inflammation, and apoptosis. Through various stimuli inside and outside of cells, ROS play a significant role as a messenger, which has attracted wide attention [46,47,48]. Studies have reported that after LPS treatment of immune cells, intracellular ROS levels are significantly increased. And ROS, as a messenger, can regulate the expression of inflammation-related factors [49,50]. In our results, we found that the expression of anti-oxidation-related factors was significantly reduced after LPS treatment, and this reaction was intensified after siGK treatment. OE GK alleviates the LPS-induced oxidative stress of KCs. These experimental results suggested that GK significantly influenced LPS-induced oxidative damage to KCs. However, whether GK can regulate the inflammatory response of cells by influencing the production of ROS and oxidative stress requires further exploration with ROS scavengers, such as NAC, after siGK treatment.

In order to further investigate the molecular mechanism of its action, this study investigated the effects of siGK and OE GK on the activation of LPS-induced inflammatory signaling pathways. Activation of PKC plays an important role in the regulation of inflammation [51,52]. The results of this study showed that the expression of PKCε in LPS-stimulated KCs by GK was the key mediator of KCs’ activation, indicating that GK may inhibit PKCε expression and have anti-inflammatory effects. MAPK is significantly activated in LPS-activated inflammatory responses [53,54]. In addition, studies have shown that PKC can significantly activate MAPK signaling pathways, and many studies have also shown that ROS can significantly activate MAPK signaling pathways [55,56]. The results of this study showed that GK influenced the expression of the p-p38 protein induced by LPS, and p38 inhibitors significantly reduced the inflammatory response exacerbated by siGK. These results suggest that the regulatory effect of GK on the LPS inflammatory response depends on the expression of p-p38. In addition, we detected the expression of the downstream factor STAT3 of MAPK [40,57,58], and found that GK significantly influenced the expression of p-STAT3, and p38 inhibitors significantly inhibited the protein expression of p-STAT3. These results suggested that GK may influence the LPS-induced inflammation by the PKCε/p38/STAT3 signaling pathway. However, studies have confirmed that PKCε has a regulatory effect on the P38 signaling pathway [59], but whether this mechanism is involved in GK-mediated inflammatory responses still needs to be further verified through experiments with PKCε inhibitors and agonists, or siRNA and an overexpression plasmid of PKCε. In addition, this study confirmed that P38 can regulate the phosphorylation level of STAT3, and the regulatory function of STAT3 in inflammatory responses has been widely supported by studies [60,61]. Furthermore, the process by which GK regulates inflammation in the liver or macrophages in animal models under different treatments may be complex. Therefore, it is necessary to further explore the protective effect of GK on LPS-stimulated inflammatory animal models and further determine the specific mechanism of GK in the inflammatory response.

**Figure 7:**
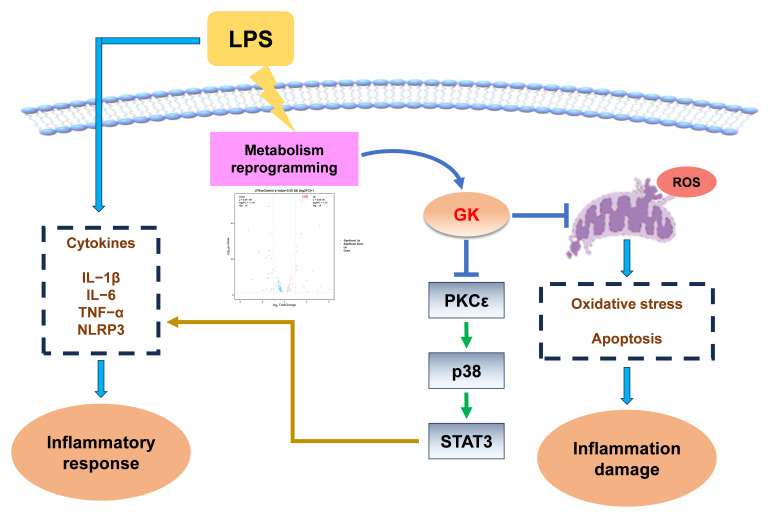
Proposed role of GK in LPS-induced Kupffer cell inflammation. GK may alleviate Kupffer cells’ inflammatory responses by inhibiting the p38/STAT3 signaling pathway and mitigating LPS-induced ROS generation.

In summary, GK may alleviate Kupffer cells’ inflammatory responses by inhibiting the p38/STAT3 signaling pathway and mitigating LPS-induced ROS generation (Figure 7). The results of this study are the first to reveal the anti-inflammatory effect of GK and its possible mechanism. The findings provide a potential reference for future development of drugs targeting GK to prevent KC inflammation and even liver damage.

## Figures and Tables

**Table 1:** Antibody information.

Antibodies	Company	Item No.
Tubulin-α	Bioworld (Nanjing, China)	BS1699
GK	Abcam (Cambridgeshire, UK)	ab228615
IL-1β	Abcam (Cambridgeshire, UK)	ab254360
NLRP3	Abcam (Cambridgeshire, UK)	ab283819
Caspase1	AdipoGen Life Sciences (San Diego, USA)	AG-20B-0042
GSDMD	Abcam (Cambridgeshire, UK)	ab219800
ASC	Cell Signalling Technology (Boston, USA)	#67824
PKCθ	Bioworld (Nanjing, China)	BS3248
PKCε	Bioworld (Nanjing, China)	BS6704
p-p38	Proteintech (Wuhan; China)	28796-1-AP
p38	Bioworld (Nanjing, China)	MB66552
p-ERK	Cell Signalling Technology (Boston, USA)	4370
ERK	Cell Signalling Technology (Boston, USA)	4695
p-JNK	Cell Signalling Technology (Boston, USA)	4668
JNK	Cell Signalling Technology (Boston, USA)	9252
p-NF-κB	Affinity (Ohio, USA)	AF2006
NF-κB	Proteintech (Wuhan; China)	10745-1-AP
p-c-Jun	Santa (Texas, USA)	sc-822
c-Jun	Affinity (Ohio, USA)	AF6090
p-STAT3	Affinity (Ohio, USA)	AF3294
STAT3	Proteintech (Wuhan; China)	10253-2-AP
Bax	Bioworld (Nanjing, China)	BS6420
Bcl2	Bioworld (Nanjing, China)	CAS7511
iNOS	Affinity (Ohio, USA)	AF0199
COX2	Abcam (Cambridgeshire, UK)	ab15191
HO-1	Proteintech (Wuhan; China)	10701-1-AP
SOD1	Bioworld (Nanjing, China)	BS6057

**Table 2:** The target genes’ primer sequences of a mouse.

Gene	Gene ID	Forward (5′ to 3′)	Reverse (3′ to 5′)
IL-6	16193	CCAAGAGGTGAGTGCTTCCC	CTGTTGTTCAGACTCTCTCCCT
IL-1β	16176	GCAACTGTTCCTGAACTCAACT	ATCTTTTGGGGTCCGTCAACT
IL-18	16173	GACTCTTGCGTCAACTTCAAGG	CAGGCTGTCTTTTGTCAACGA
TNF-α	21926	GACGTGGAACTGGCAGAAGAG	TTGGTGGTTTGTGAGTGTGAG
iNOS	18126	GTTCTCAGCCCAACAATACAAGA	GTGGACGGGTCGATGTCAC
COX2	19225	TTCAACACACTCTATCACTGGC	AGAAGCGTTTGCGGTACTCAT
GK	14933	TGAACCTGAGGATTTGTCAGC	CCATGTGGAGTAACGGATTTCG
PPIA	268373	GGGTTCCTCCTTTCACAGA	CCATCCAGCCATTCAGTC
NQO1	18104	AGGATGGGAGGTACTCGAATC	AGGCGTCCTTCCTTATATGCTA
SOD1	20655	AACCAGTTGTGTTGTCAGGAC	CCACCATGTTTCTTAGAGTGAGG
HO-1	15368	AAGCCGAGAATGCTGAGTTCA	GCCGTGTAGATATGGTACAAGGA
GPX1	14775	AGTCCACCGTGTATGCCTTCT	GAGACGCGACATTCTCAATGA
NRF2	18024	TCTTGGAGTAAGTCGAGAAGTGT	GTTGAAACTGAGCGAAAAAGGC
GST1	56615	ATGCCACCATACACCATTGTC	GGGAGCTGCCCATACAGAC

## Data Availability

The original contributions presented in this study are included in the article. Further inquiries can be directed to the corresponding author.

## Abbreviations

The following abbreviations were used in this manuscript:

Bax	Bcl2-associated x protein
Bcl2	Apoptosis regulator Bcl-2
COX2	Cyclooxygenase2
c-Jun	Jun activation domain-binding protein
ERK	Extracellular-regulated protein kinase
GK	Glycerol kinase
HO-1	Heme oxygenase 1
IL	Interleukin
iNOS	Inducible nitric oxide synthase
JNK	Stress-activated protein kinase 1
LPS	Lipopolysaccharide
NLRP3	NOD-like receptor pyrin domain-containing protein 3
NF-κB	Nuclear factor kappa-B
p38	Cytokine-suppressive anti-inflammatory drug-binding protein
PI	Propidium iodide
SOD1	Superoxide dismutase 1
PKC	Protein kinase C
ROS	Reactive oxygen species
STAT3	Signal transducer and activator of transcription 3
